# Integrated multi-omics analysis of ovarian cancer using variational autoencoders

**DOI:** 10.1038/s41598-021-85285-4

**Published:** 2021-03-18

**Authors:** Muta Tah Hira, M. A. Razzaque, Claudio Angione, James Scrivens, Saladin Sawan, Mosharraf Sarker

**Affiliations:** 1 School of Health and Life Sciences, Teesside University, Middlesbrough, TS4 3BX UK; 2 School of Computing, Eng. & Digital Tech., Teesside University, Middlesbrough, TS4 3BX UK; 3 The James Cook University Hospital, Middlesbrough, TS4 3BW UK

**Keywords:** Cancer, Mathematics and computing

## Abstract

Cancer is a complex disease that deregulates cellular functions at various molecular levels (e.g., DNA, RNA, and proteins). Integrated multi-omics analysis of data from these levels is necessary to understand the aberrant cellular functions accountable for cancer and its development. In recent years, Deep Learning (DL) approaches have become a useful tool in integrated multi-omics analysis of cancer data. However, high dimensional multi-omics data are generally imbalanced with too many molecular features and relatively few patient samples. This imbalance makes a DL based integrated multi-omics analysis difficult. DL-based dimensionality reduction technique, including variational autoencoder (VAE), is a potential solution to balance high dimensional multi-omics data. However, there are few VAE-based integrated multi-omics analyses, and they are limited to pancancer. In this work, we did an integrated multi-omics analysis of ovarian cancer using the compressed features learned through VAE and an improved version of VAE, namely Maximum Mean Discrepancy VAE (MMD-VAE). First, we designed and developed a DL architecture for VAE and MMD-VAE. Then we used the architecture for mono-omics, integrated di-omics and tri-omics data analysis of ovarian cancer through cancer samples identification, molecular subtypes clustering and classification, and survival analysis. The results show that MMD-VAE and VAE-based compressed features can respectively classify the transcriptional subtypes of the TCGA datasets with an accuracy in the range of 93.2-95.5% and 87.1-95.7%. Also, survival analysis results show that VAE and MMD-VAE based compressed representation of omics data can be used in cancer prognosis. Based on the results, we can conclude that (i) VAE and MMD-VAE outperform existing dimensionality reduction techniques, (ii) integrated multi-omics analyses perform better or similar compared to their mono-omics counterparts, and (iii) MMD-VAE performs better than VAE in most omics dataset.

## Introduction

Ovarian cancer is a common and deadly gynaecological cancer with a high mortality rate in developed countries. It accounts for 5% of all cancer deaths in females in the UK^1^ and USA^2^. Ovarian cancers are generally diagnosed at an advanced age as the early-stage disease is usually asymptomatic, and symptoms of the late-stage disease are nonspecific^3^. The anatomical location and the ovaries’ position are mainly responsible for asymptomatic and nonspecific nature of the disease. Since the ovaries have limited interference with the surrounding structures, ovarian cancer is hard to detect until the ovarian mass is significant, or metastatic disease supervenes. Due to the symptoms’ nonspecific nature, it often requires multiple consultations with a primary care physician and several investigations in finding the disease and for an appropriate therapy/treatment. In this context, the development of useful tools to better understand the complex pathogenesis is needed for effective cancer management and prognosis^3–5^.

Cancer is a complex and heterogeneous disease that deregulates cellular functions in different molecular levels, including DNA, RNA, proteins and metabolites. Importantly, molecules from different levels are mutually associated in reprogramming the cellular functions^6–8^. Any study limited to any of these levels is insufficient to understand the complex pathogenesis of cancer. Integrated multi-omics analysis of data from these levels is essential to understand cellular malfunctions responsible for cancer and its progression holistically. Importantly, integrated multi-omics analysis, taking the advantage of various omic technologies (i.e., genomics, transcriptomics, epigenomics and proteomics), can identify reliable and precise biomarkers for diagnosis, treatment stratification and prognosis^5,9^. Recent advancements of omics and computational technologies, including deep learning, have boosted the research in integrated multi-omics analysis for precision medicine and cancer. In recent years, many research works^10–12^ have been published on integrated multi-omics analysis of cancer. These works are either on individual cancer^13–18^ or pancancer^19–22^. Most of these works integrated di-omics^15,16^, few of them integrated tri-omics^23^, and very few of them integrated tetra-omics^24^ data. There are a few integrated multi-omics analyses of ovarian cancer, including di-omics^15,25^ and tri-omics^13,25–27^ based analyses.

Due to the increasing availability of large-scale multi-omics cancer data, machine learning approaches, especially DL approaches^16,18,28,29^ are becoming very useful and effective in integrated multi-omics analysis of cancer data. However, high dimensional omics data are normally imbalanced with a large number of molecular features and a relatively small number of available samples with clinical labels^29^. For example, DNA methylation and mRNA integrated dataset (TCGA) for ovarian cancer has 39,622 features (27,579 features for DNA methylation and 12,043 features for mRNA) for common 481 samples with clinical labels. This imbalanced dimensionality of cancer datasets makes it challenging to use a machine learning (ML) or DL in integrated multi-omics analysis, especially in individual cancers as they have few samples. For instance, TCGA ovarian cancer RNAseq dataset has only 308 samples, whereas pancancer RNAseq dataset has 9081 samples ^29^. Algorithms for dimensionality reduction, such as autoencoder-based DL algorithms^16,18^, together with conventional solutions, such as principal component analysis (PCA)^30^, are possible solutions to the dimensionality problem. Importantly, considering the discontinuous and non-generative nature of traditional autoencoders, VAEs^31^ have emerged as DL-based generative models for compressed features learning or dimensionality reduction. There are many works which have used VAE in their studies. However, they are mostly mono-omics studies of individual cancer^28,32,33^ or pancancer^29,34,35^. OmiVAE^29^ is the only work that considered VAE for integrated multi-omics (di-omics) analysis of pancancer. Also, VAE may suffer in representing the input features due to uninformative compressed features and variance over-estimation in feature space^36^. None of the existing works has used MMD-VAE, and also their analyses are limited to cancer molecular subtypes classification only.

Moreover, most of the existing works^28,32,33,35^ use unsupervised dimensionality reduction methods, separating the downstream analysis from the reduction method. However, dimensionality reduction in cancer multi-omics analysis is an intermediate step toward the downstream analysis, like classification (e.g., cancer vs normal cell). Separating the dimensionality reduction and model (e.g., a classifier) learning may not be optimal for classification as datasets are not always suitable for a classification task. For example, a DNA methylation dataset that includes cancer and normal samples is readily applicable for classification due to their discriminative features. However, a DNA methylation dataset that only includes cancer samples may not be useful in classification. Due to lack of supervision during a dimensionality reduction process, some key features can be filtered before training the classifier, affecting the final performance^29,37,38^. In this context, supervised dimensionality reduction methods can be more useful in balancing multi-omics datasets and their integrated downstream analysis.

In this work, we did an integrated multi-omics analysis of ovarian cancer using VAE and MMD-VAE^36^. To the best of our knowledge, this is the first work that does a comprehensive mono-omics and integrated multi-omics (i.e., di- and tri-omics) analysis of individual cancer (ovarian) using VAE or MMD-VAE, or both. The objectives of this work are three-fold. First, we have designed and developed a DL architecture of VAE and MMD-VAE that supports unsupervised and supervised learning of latent features from mono-omics, di-omics and tri-omics data. Second, we did a dimensionality reduction performance analysis of the developed DL architecture on ovarian cancer by cancer samples identification, molecular subtypes clustering and classification. As a dimensionality reduction technique, the performance of MMD-VAE or VAE depends on input features and sample size, not on the cancer type. Hence, we have tested the developed MMD-VAE and VAE using datasets with three different sample sizes (i.e., 292, 459 and 481) to demonstrate our findings’ robustness. Finally, a survival analysis of an existing ovarian cancer dataset has been carried out using the reduced or latent features sets.

## Methods

In the following, we briefly discuss the datasets used, data preprocessing, VAE/MMD-VAE architecture, dimensionality reduction and survival analysis methods.

### Datasets used

We used mono-omics and multi-omics (i.e., di- and tri-omics) data for the study. We generated multi-omics data using different combinations of high dimensional mono-omics data. Table 1 summarises the datasets used in this study in terms of their key features (i) omic-count (mono/di/tri), (ii) omic type (e.g.,genomics, epigenomics, transcriptomics, and their combinations) (iii) omic data (i.e., mRNA, CNV/CNA, DNA methylation, RNAseq, and miRNA) (iv) input features dimension, (v) sample size (after processing), and (vi) the unit used for data values (e.g., beta value for DNA methylation). Here, CNV/CNA means copy number variation/alteration, mRNA means gene expression array, DNA methylation means methylation of CPG islands, and RNAseq means gene expression by RNAseq. We have downloaded four mono-omics TCGA datasets from UCSC Xena data portal^39^, one for mRNA, CNV/CNA and RNAseq, and two for DNA methylation. All the mono-omics datasets except the second DNA methylation dataset are from the TCGA Ovarian Cancer (OV) cohort^40–43^. The second DNA methylation dataset is from GDC TCGA Ovarian Cancer (OV) cohort^44^. It includes cancer and normal samples. We have concatenated these mono-omics data to form the di-omics and tri-omics datasets. The table’s ’Feature dimension’ and ’Sample size’ columns demonstrate that all the datasets are imbalanced with too many input features and relatively too few numbers of samples with clinical labels. For example, one tri-omics (CNV + DNA methylation + RNAseq) dataset has 72,885 input features with only 292 samples.

**Table 1:** Key features of the datasets used.

Omic count	Omic type	Omic data	Feature size	Sample size (${}^{1}$)	Feature & Data values (unit)	Source
mono-omics	Genomics (G)	CNV	24,776	481	Gene name & Gistic2 copy number	^41^
	Transcriptomics (T)	mRNA	12,043	481	Gene name & log2(affy RMA)	^40^
		RNAseq	20,530	292	Gene name & pan-cancer normalized log2(norm_count+1)	^42^
	Epigenomics (E)	DNA methylation	27,579 / 21,675	481/886	CPG probe identifier or CG number & Beta value	^43,44^
Di-omics	G + T	CNV + mRNA	36,819	481	A combination of the respective mono-omics values	${{}^2}$
		CNV+ RNAseq	45,306	292		
	E + T	DNA methylation + mRNA	39,622	481		
		DNA methylation + RNAseq	48,109	292		
	G + E	CNV+ DNA methylation	52,355	481		
Tri-omics	G + E + T	CNV+ DNA methylation + mRNA	64,398	481		
		CNV+ DNA methylation + RNAseq	72,885	292		

${}^1$Sample size after intersection.

${}^2$Generated through the concatenation of respective mono-omics datasets.

### Data prepossessing

The downloaded datasets are not ready (e.g., sample sizes are not equal- CNV dataset has 579 and mRNA dataset has 593 samples) to be used in dimensionality reduction and integrated multi-omics analysis. They need to be preprocessed, such as the datasets’ sample sizes need to be same to integrate and generate di- and tri-omics datasets. We have preprocessed the downloaded datasets (TCGA Ovarian Cancer cohort) using a four steps method (see Fig. 6 in Supplementary documents).

- *Step 1* First, we intersected the mono-omics datasets to find the common and same size samples. We did two different intersections of the datasets (Fig. 6 (step: 1) in Supplementary documents) using common sample IDs (also represent the patient IDs) to keep the maximum number of samples for the study. The intersection of CNVs, mRNA and DNA methylation datasets has found 481 samples, and the intersection of CNVs, DNA methylation and RNAseq has found 292 samples in common within the datasets.
- *Step 2* We identified and removed the missing/zero/NA values in the four downloaded omics files. All the data files, except the RNAseq, had no missing/zero/NA values, and 212 input features or genes (particularly small nucleolar RNA/SNORD) with zero expression values were removed from the RNAseq dataset.
- *Step 3* Non-normalised datasets, such as CNVs and RNAseq datasets, were normalised using the min-max technique. We used the min-max normalisation as unlike other techniques (i.e., Z-score normalisation) it guarantees multi-omics features will have the same scale^45^. Thus, all the features will have equal importance in the multi-omics analysis.
- *Step 4* Finally, we concatenated the normalised mono-omics datasets to form the di- and tri-omics datasets. Concatenations involving RNAseq, such as $$CNVs + RNAseq, DNA methylation + RNAseq $$ and $$CNVs + DNA methylation + RNAseq$$ datasets have 292 samples and others have 481 samples.

The second DNA methylation^44^ dataset is highly imbalanced (Fig. 8a in Supplementary documents) as it has only 10 normal samples compared to the 603 cancer samples (class ratio: 1.36:98.64). We used the Borderline-SMOTE SVM^46^ to reduce the class imbalance (Fig. 8b in Supplementary documents) by re-sampling of normal samples (10 to 283). After the re-sampling, we have 886 samples compared to the original 613 samples. This has increased class ratio (31.94:68.06) between the normal and cancer samples.

### VAE/MMD-VAE architecture

#### Standard VAE

A VAE^31^ is a deep generative model, which can learn meaningful data manifold from high dimensional input data. Unlike, standard autoencoders (Fig. 7 in Supplementary documents), a VAE encodes an input ($x^i$) as a distribution over a latent space instead of as a single point. Given an omic/multi-omics dataset *D* with *N* samples $\{x^{i}\}_{i= 1}^N$ with *d* dimensional omic or multi-omics features, a VAE/MMD-VAE assumes each sample $x^i\in {\mathbb {R}}^d$ is generated from a latent vector $z^i\in {\mathbb {R}}^p$, where $d\gg p$. A DL model for VAE follows a four-step process:

- Step 1 (encoding): an encoder encodes or generate each latent variable $z^i$ from a prior distribution or latent distribution $p_{\theta }(z)$. Importantly, the encoder introduces a variational distribution $q_{\phi } (z|x)$ (also known as encoding distribution) to estimate the posterior and address the intractability of the true posterior $p_\theta (z|x)$ in calculating the distribution of *X* or $p_{\theta }(X)$^31^. Here, $\phi$ is the set of learnable parameters of the encoder.
- Step 2 (sampling): a sampler samples points from the latent space by sampling from the encoded or encoding distribution $q_{\phi } (z|x)$.
- Step 3 (decoding) : a decoder decodes the sampled points from a conditional distribution $p_{\theta }(x|z)$ and reconstructs the inputs $x^\prime$, where $\theta$ is the set of learnable parameters of the decoder. In this step, VAE also calculates the loss or error using loss function that is composed of a reconstruction term and a regularisation term. The reconstruction term calculates the reconstruction loss and the regularisation term quantifies the distance between the estimated posterior $q_{\phi } (z|x)$ and true posterior $p_\theta (z|x)$ to regularise the latent space. A standard VAE uses Kullback-Leibler divergence^47^ for the regularisation term and jointly optimises the encoder and decoder using the following loss function that rely on the traditional evidence lower bound (ELBO) criterion:
  1 $$\begin{aligned} L_{VAE} = {\mathbb {E}}_{q_{\phi }(z|x)}[\log p_{\theta }(x|z)] - D_{KL}(q_{\phi } (z|x)||p_{\theta }(z)) \end{aligned}$$
  where $D_{KL}$ is the Kullback-Leibler (KL) divergence between two probability distributions.
- Step 4 (backpropagation): finally, the calculated loss is backpropagated through the network to update the model accordingly.

**Figure 1:**
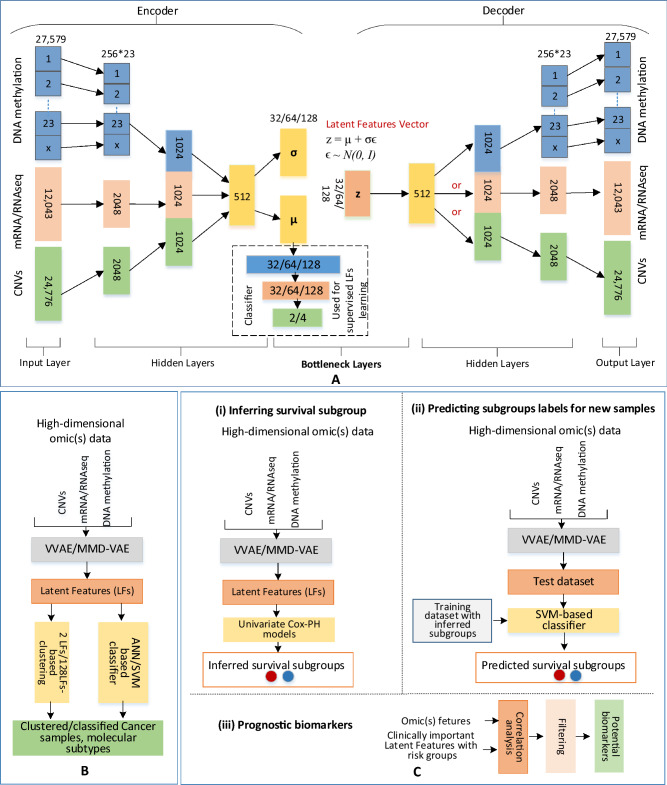
Methods: (**A**) VAE/MMD-VAE architecture consists of an encoder and a decoder made from 3 hidden layers and a bottleneck made from 2 layers and a 3-layered ANN-based classifier for supervised LFs learning, (**B**) Clustering using 2 LFs and ANN-based classification (e.g., cancer vs normal, and molecular subtypes) using 2 and 128 LFs, (**C**) Survival analysis using 128 LFs: (i) inferring survival subgroup, (ii) predicting subgroup and (iii) potential prognostic biomarkers.

#### MMD-VAE

VAE using ELBO-based loss function (Eq. 1) may suffer from the following two issues^36^:

- Uninformative latent code/feature: The regularisation term ($D_{KL}(q_{\phi } (z|x)||p_{\theta }(z))$ used in the loss function (Eq. 1) might be too restrictive^48,49^. KL divergence naturally encourages the latent code $q_{\phi } (z|x)$ to be a random sample from $p_{\theta }(z)$ for each *x*, making the code uninformative/unaware about the input. In this context, the encoder could fail to learn any meaningful latent representation of the input.
- Overestimation of variance in feature space: The ELBO-based VAE tends to over-fit data. Due to the over-fitting, it could learn a $q_{\phi } (z|x)$ that has variance tending to infinity^36^. For example, training ELBO-based VAE on a dataset with two data points $\{ 2, -2\}$, and both encoder ($q_{\phi } (z|x)$) and decoder ($p_\theta (x|z)$) output Gaussian distributions with non-zero variance.

Use of Maximum Mean Discrepancy (MMD) in loss function instead of the $D_{KL}$^50^ can address the above issues. According to the MMD, two distributions are identical if and only if all of their moments are same. Unlike $D_{KL}$, MMD-based regularisation term estimate divergence by how “different” the moments of two distributions *p*(*z*) and *q*(*z*) are. We can use the kernel embedding trick to estimate MMD for two distributions as Eq. (2).

2 $$\begin{aligned} MMD(p(z)||q(z)) = {\mathbb {E}}_{p(z),p(z^\prime )}[k(z, z\prime )] + {\mathbb {E}}_{q(z),q(z^\prime )}[k(z, z^\prime )] - 2 {\mathbb {E}}_{p(z),q(z^\prime )}[k(z, z^\prime )] \end{aligned}$$

where $k(z, z^\prime )$ is any universal kernel, including Gaussian kernel $k(z, z^\prime ) = e^{-}\frac{\parallel z-z^\prime \parallel ^2}{2\sigma ^2}$. The VAE using the MMD-based loss function is known as MMD-VAE, and the corresponding loss function for can be expressed as Eq. (3).

3 $$\begin{aligned} L_{MMd-VAE} = {\mathbb {E}}_{q_{\phi }(z|x)}[\log p_{\theta }(x|z)] + MMD(q_{\phi } (z|x)||p_{\theta }(z)) \end{aligned}$$

#### VAE/MMD-VAE architecture

As VAE and MMD-VAE differ only in the loss function, we can implement them using the same architecture. The implementation presented in Fig. 1 can support unsupervised and supervised dimensionality reductions. The architecture includes three main components: an encoder, a decoder and a classifier. For the unsupervised dimensionality reduction, the encoder and the decoder learn latent features from the input without the classifier’s support. However, we need the classifier for supervised learning of latent features.

Like other deep neural network architectures, VAE has two main hyperparameters: the number of layers and the number of nodes in each hidden layer. Systematic experimentation is the most reliable way to configure this hyperparameters^51^. We used the configurations from an existing and related work OmiVAE^29^ to avoid the experimentation from scratch. We used the same number of hidden layers as the OmiVAE and ran a few experiments to identify the suitable nodes for the hidden and bottleneck layers. For example, we experimented with the hidden layer one of the encoder and decoder with 4096 and 2048 nodes. We selected 2048 due to insignificant performance difference between the two sizes and shorter processing time for 2048 nodes.

The encoder network comprised of an input layer and three hidden layers. The decoder network structure is the mirror image of the encoder structure. Notably, the encoder and decoder share the necessary bottleneck layers. We used the architecture for mono-omics, di- and tri-omics data, and the size of the bottleneck layers is same for all the datasets. However, the other layers’ sizes varied according to the omic count (i.e., mono, di and tri) and omic data. For example, for mono-omics data, such as mRNA data, the input and output layers are 12,043, and hidden layers sizes are 2048 and 1024. As shown in Fig. 1, multi-omics data were integrated using an unsupervised parallel integration method^12^. The classifier used a 3-layered fully connected artificial neural network (ANN) with an input layer with nodes equal to the LFs (32/64/128), a hidden layer with nodes equal to the half of LFs, and an output layer with nodes equal to the class numbers (e.g., 2 for cancer vs normal samples, 4 for molecular subtypes).

The VAE/MMD-VAE architecture does all the activities illustrated in Fig. 1. In the following, using omic(s) data, we briefly discuss these activities in the perspective of the encoder, decoder and classifier.

- Encoder: The encoder network using two hidden layers encodes mono-omics data into a 1024 dimensional vector, di-omics data into two 1024 dimensional vectors and tri-omics data into three 1024 dimensional vectors. The encoding network for the DNA methylation data is different from the other omics data. For example, in the first hidden layer, each chromosome related DNA methylation data are encoded into corresponding vectors with 256 dimensions wheres for the others, input data are encoded into a 2048 dimensional vector. This encoding is to capture the intra-chromosome relationships, and second hidden layer for the DNA methylation data captures the inter-chromosome relationships. For di- and tri-omics data, the second hidden layer respectively concatenates two and three 1024 dimensional vectors and produces an encoded 512-dimensional vector. The encoder’s final hidden layer fully connects to two output layers. These two layers of the size of latent code or features (32/64/128) are part of the bottleneck layers and represent the mean $\mu$ and the standard deviation $\sigma$ in the Gaussian distribution $N(\mu , \sigma )$ of the latent variable or feature *z* given input sample *x* or simply $q_{\phi } (z|x)$. As illustrated in Figs. 1 and 7 in Supplementary documents, a reparameterisation trick is applied ($z = \mu + \sigma \epsilon$, where $\epsilon$ is a random variable sampled from unit normal distribution *N*(0, *I*)) in the bottleneck layer to make the sampling process differentiable and suitable for backpropagation. The sampled latent features vector (z/LFs) is the compressed lower-dimensional representation of omics or integrated multi-omics data.
- Decoder: The decoder network takes the latent feature vector *z* as the input and passes through three hidden layers, and finally outputs the reconstructed vector $x^\prime$ of the input omics data. The decoder is also responsible for estimating the overall loss using Eqs.  (4) and (6) respectively for VAE and MMD-VAE.
  4 $$\begin{aligned} L_{VAE} = k\frac{1}{M}\Sigma _{j=1}^M CE(x_{m_{j}}, x^\prime _{m_{j}}) + \Sigma _{c=0}^c CE(x_{om_{c}}, x^\prime _{om_{c}}) + L_{KL} \end{aligned}$$
  where k is a binary variable set to 1, if there is any DNA methylation data in the input otherwise set to 0, *M* is the number of chomosomes, CE is the binary cross-entropy between input data (i.e., $x_{m_{j}}$ - DNA methylation, $x_{om_{c}}$ - other omic data) and reconstructed data (i.e., $x^\prime _{m_{j}}$ - DNA methylation and $x^\prime _{om_{c}}$ - other omic data), $c = 0, 1, 2$ - other omic data count, and $L_{KL}$ is the KL divergence between the learned distribution and a unit normal distribution *N*(0, *I*), which is:
  5 $$\begin{aligned} L_{KL}= & {} D_{KL}(N(\mu , \sigma )\parallel N(0, I)) \end{aligned}$$
  6 $$\begin{aligned} L_{MMD-VAE}= & {} k\frac{1}{M}\Sigma _{j=1}^M nll(x_{m_{j}}, x^\prime _{m_{j}}) + \Sigma _{c=0}^c nll(x_{om_{c}}, x^\prime _{om_{c}}) + L_{MMD} \end{aligned}$$
  where *nll*- negative log likelihood which can be calculated as mean of $(x_{m_{j}}- x^\prime _{m_{j}})^2$ for DNA methylation data and mean of $(x_{om_{c}}- x^\prime _{om_{c}})^2$ for other omics data, $L_{MMD}$ is MMD (Eq. 2) between the learned distribution and a unit normal distribution *N*(0, *I*), which is:
  7 $$\begin{aligned} L_{MMD} = MMD(N(\mu , \sigma )\parallel N(0, I)) \end{aligned}$$
  Classifier: In an unsupervised VAE, the bottleneck layer tends to extract the essential features to reconstruct input samples as closely as possible. However, these extracted features may not be related to a specific task, such as a molecular subtype classification. The classifier works as an additional regularisation on top of the bottleneck layer. With this additional regularisation, the classifier encourages the VAE or MMD-VAE network to learn LFs that can not only accurately reconstruct the input sample but also, identify cancer and classify molecular subtypes^29^. The binary cross-entropy based classification loss ($L_{cl}$) can be added to $L_{MMD-VAE}$ or $L_{VAE}$ to estimate the total loss using the following Equation:
  8 $$\begin{aligned} L_{{VAE}_{total}} = \alpha L_{VAE} + \beta L_{cl} \end{aligned}$$
  where $\alpha$ and $\beta$ are weights of the two losses in the total loss. Equation (8) can be used for the total loss of MMD-VAE ($L_{{MMD-VAE}_{total}}$) by replacing $L_{VAE}$ with $L_{MMD-VAE}$. The supervised and unsupervised learning of LFs depends on the value of $\beta$. We use $\beta = 0$ for the unsupervised and $\beta = 1$ or any positive value for the supervised learning of LFs.

We used a batch normalisation technique in each fully connected block to implement the VAE/MMD-VAE DL architecture. This is to address the *internal covariate shift* (The distribution of the inputs of each layer changes during training, when previous layers’ parameters change, which slows down the training process.)  by normalising layer inputs^52^. Thus, it stabilises the learning process and significantly improves the learning speed. As the activation function, we used the rectified linear units (ReLU) for the hidden layers, the sigmoid for the decoder’s output layer and the softmax for classifiers’ output layer. We built the model using PyTorch (version 1.5.0). The implementations of the models used in this paper are available on GitHub (https://github.com/hiraz/MMD-VAE4Omics).

#### Clustering and classification in cancer

Cancer samples identification and molecular subtypes are useful in prognostic and therapeutic stratification of patients and improved management of cancers^13,25,53^. Hence, correct clustering and classification of ovarian cancer samples and molecular subtypes are important for improved disease management. Authors in^13,25,53^ have identified four ovarian cancer transcriptional (one molecular subtype (We will use transcriptional subtypes and molecular subtypes interchangeably.)) subtypes, which may have clinical significance. These four subtypes of high grade serous ovarian cancer (HGS-OvCa) are named as Immunoreactive, Differentiated, Proliferative and Mesenchymal^13,54^. The datasets used in this work are about HGS-OvCa, and the clinical data include these molecular subtypes for most of the samples. Although these molecular subtypes are transcriptional (e.g., mRNA), they can be used for other omics data analysis due to their correlation or association with transcriptional data^55–58^. For example, authors in^55^ have reported that DNA methylation is often negatively associated with gene expression in promoter regions, while DNA methylation is often positively associated with gene expression in gene bodies.

VAE or MMD-VAE generated latent and compressed features (*z* or LFs) can be used to cluster and classify cancer samples, subtypes, including existing transcriptional or molecular subtypes of ovarian cancer. The performance of clustering and classification exploiting *z* can demonstrate the dimensionality reduction capability of VAE or MMD-VAE. We demonstrated the dimensionality reduction capability of VAE and MMD-VAE using the latent features learned from the mono-omics, integrated di-and tri-omics data of ovarian cancer, and used for the followings:

- *Clustering* We can use the *LFs* learned (unsupervised and supervised) by the VAE/MMD-VAE models to cluster samples into cancer vs normal and molecular subtypes. We used a two- and three-dimensional embedding of the mono-omics, di- and tri-omics features for the selected samples, and visualised the clustered samples using scatter plots. Two dimensional (2D) and three dimensional (3D) embedding of the omic(s) features for the selected samples are accomplished by selecting first 2 and 3 LFs from the learned LFs (Fig. 1B (left-side)). We then used the embedded features to cluster the samples into two groups for cancer identification (cancer and normal samples), and four groups (4 molecular subtypes) for molecular subtypes using 2D and 3D scatter plots.
- *Classification* We used an ANN-based classifier to classify cancer samples and molecular subtypes using the LFs learned through the unsupervised process. For all the omics data, we selected the first two and all LFs learned by VAE/MMD-VAE to classify the samples (Fig. 1B right-side). For the LFs learned through the supervised process, we used the VAE/MMD-VAE architecture’s classifier to classify the molecular subtypes. All the classification experiments were validated using a 5-fold cross-validation. In each round of the validation, 80% data were used for the training, and the rest 20% were left out from the training and used for separate testing. We presented the classification performances for both classifiers in terms of accuracy, precision, recall, and f1 score. We also presented a confusion matrix for each classification task done using the LFs learned through supervised VAE/MMD-VAE models. We have selected the first 2 LFs for the clustering and classification for simplicity reason. However, one can select any 2 LFs from the learned LFs, and the performance will be similar to the presented ones.

For the LFs learned using the unsupervised VAE/MMD-VAE models, we compared the clustering and classification performances with two popular traditional dimensionality reduction methods, namely PCA and t-SNE^59^. We also illustrated how a combination of a traditional method (e.g., t-SNE) and MMD-VAE/VAE performs in molecular subtypes clustering.

#### Survival analysis

Identification of robust survival subgroups of ovarian cancer (HGS-OvCa) can significantly improve patient care. Existing molecular subtypes of HGS-OvCa, such as transcriptional molecular subtypes^13^ may not be useful in survival subgroups prediction as most of these studies do the subtyping without relying on survival data. In this study, first, we used existing transcriptional subtypes for survival analysis and then used the learned (supervised) LFs inferring and predicting survival subgroups of HGS-OvCa. We followed a 3-step process (Fig. 1C) as below to do the subgrouping and their corresponding survival analysis:

- Inferring survival subgroup: We built a univariate Cox proportional hazards (Cox-PH) model for each of the LFs produced by the VAE/MMD-VAE (Fig. 1C(i)). Then, we identified clinically relevant LFs for which a significant Cox-PH model was found (log-rank $p <0.05$). Next, we used these reduced and clinically relevant LFs (CRLFs) to cluster the samples using a K-means clustering algorithm. We used the R package NbClust^60^ to determine the optimal *K* value (number of clusters). NbClust can calculate up to 30 indices or metrics to determine the optimal number of clusters in a data set. It also identifies the best value for *K* by the majority rule. In our all 11 datasets (see Table 1), optimal values were between 4 and 2. Considering the small sample sizes 481 and 292 with a low number of events, we chose $K = 2$, which means we identified/inferred two survival subgroups.
- Predicting survival group labels for new samples: After having the survival subgroups labels from K-means clustering, we used an SVM-based classifier (Fig. 1C(ii)) to predict survival subgroup labels for new samples. We used a 60%/40% (training/test sets) of all the datasets to have sufficient test samples in most cases that generate evaluation metrics. We used the *tune* function of R package *e*1071^61^ to train the SVM model as it tunes the model parameters through cross-validation (5-fold) and identify the best model for a training dataset. In each round of the validation, 60% data were used for training and rest 40% were left out from the training as the test dataset. Finally, we used the test dataset to predict the survival subgroup or risk labels. We used the Cox-PH model and Kaplan-Meier (KM) survival curves to evaluate survival prediction performance. We used the following three metrics for the evaluation:
  *Concordance index* The concordance index or C-index is a metric to evaluate the predictions made by an algorithm. Based on Harrell C statistics^62^, C-index can be defined as the fraction of all pairs of individuals whose predicted survival times are correctly ordered^63^. The C-index score range between 0 and 1 and a score around 0.70 indicates a good model, whereas a score around 0.50 means predictions are no better than a coin flip in determining which patient will live longer. To calculate the C-index, we first built a multi-variate Cox-PH model using the training set (including the inferred survival subgroup labels and clinical features). We then predicted survival using the labels of the test set. We then computed the C-index using *concordance* function of R’s *survival* package^64^. Similarly, we calculated the C-index only considering the clinical features (i.e., status, grade).
  *P value of Cox-PH regression* The Cox-PH models built on training datasets compute log-rank p values for the models. Also, we plotted the Kaplan-Meier survival curves of the two survival subgroups (predicted) and calculated the log-rank p-value of the survival difference between them.
  *Brier score* Brier score function measures the accuracy of probabilistic prediction^65^. In survival analysis, it measures the mean of the difference between the observed and the estimated survival beyond a certain time^66^. The score ranges between 0 and 1, and a smaller score indicates better accuracy of the prediction. We used the R Package *survAUC*^67^ to compute the Brier score.
- Identifying prognostic biomarkers from LFs: We did all the above analyses using the LFs as their compressed representations simplify the survival analysis and molecular subtyping. However, we need to map these LFs back to their corresponding input features to identify potential molecular biomarkers. We mapped the associated input features for each clinically relevant LF using a linear model and filtered the features with zero or insignificant input feature values. Next, we estimated the correlation between the CRLFs and their corresponding input features. Finally, we used the filtered correlation data for hierarchical clustering (colour map) of LFs and their input features.

## Results

We used the developed DL architecture of VAE/MMD-VAE for cancer samples identification, molecular subtypes clustering and classification, and survival analysis using the TCGA ovarian cancer datasets. The results demonstrate the performance of the VAE and MMD-VAE in dimensionality reduction and survival analysis.

We trained and tested the developed VAE and MMD-VAE models with three different bottleneck layers ($LFs/z = 32, 64, 128$) on the preprocessed omics datasets to demonstrate the integrated multi-omics data analysis capability. We implemented the DL model of VAE/MMD-VAE using the network architecture presented in Fig. 1. We tested the model in unsupervised and supervised settings. We used the Adam optimiser with learning rate $10^{-3}$ due to its superior performance compared to other stochastic optimisation methods^68^. We reported the results only for $LFs/z = 128$ due to space limitation and a similar performance pattern. All the classification performances were cross-validated. Two sets of results were generated, one on cancer samples identification and molecular subtypes clustering and classification, and another on survival analysis. Importantly, we ran the experiments on four mono omics, five di-omics and two tri-omics datasets’. We presented the results for only one for each omics data due to space limitation.

### Dimensionality reduction

We have demonstrated the dimensionality reduction capability of the developed VAE/MMD-VAE by ovarian cancer samples identification, and molecular subtypes clustering and classification. We also carried out a survival analysis of the TCGA ovarian cancer dataset with the latent features set.

#### Clustering

**Figure 2:**
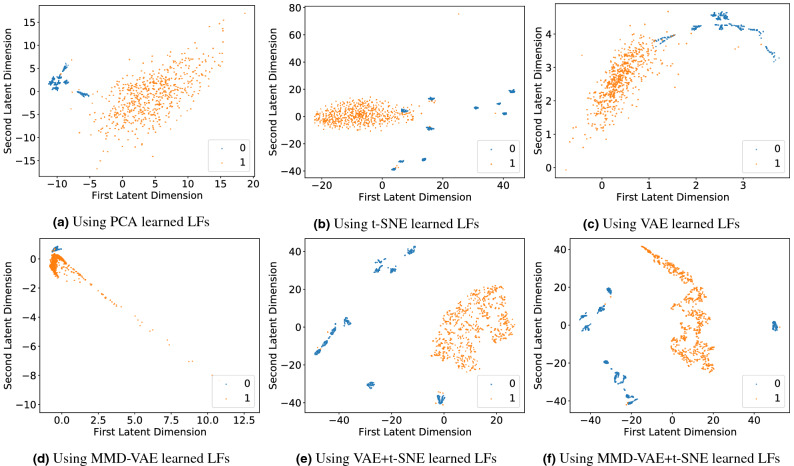
Clustering of normal and cancer samples using the LFs learned using unsupervised PCA, t-SNE, VAE & MMD-VAE (using 2D for PCA & t-SNE and first 2 LFs for VAE and MMD-VAE) (**a**)–(**d**)) on DNA methylation (mono-omics) data from the GDC cohort. t-SNE was used (**e**,**f**) on the 128 learned LFs to identify 2 LFs for the clustering. Legends: 0—Normal, 1—Cancer.

**Figure 3:**
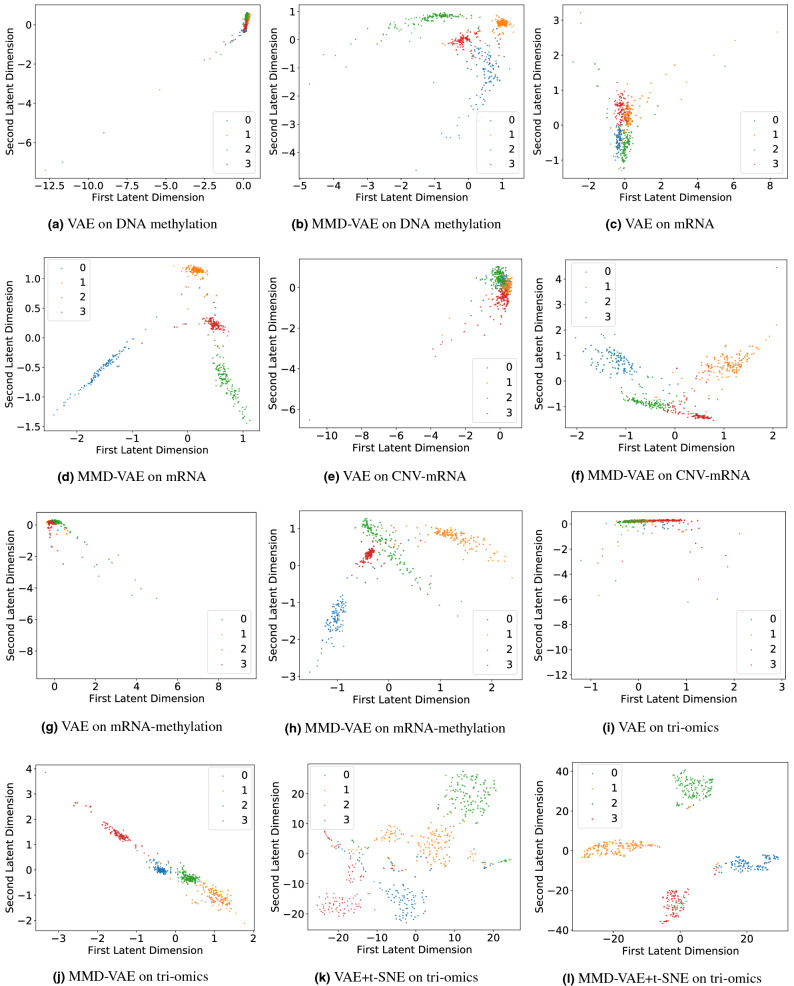
Clustering molecular subtypes using the LFs learned through the supervised VAE & MMD-VAE + t-SNE (2D or 2 LFs): (**a**–**c**) for MMD-VAE respectively for mono-omics, di-omics and tri-omics data, (**d**–**f**) for MMD-VAE + t-SNE respectively for mono-omics, di-omics and tri-omics data. Legends: 0—Immunoreactive, 1—Differentiated, 2—Proliferative and 3—Mesenchymal.

- Cancer vs Normal samples: We used the unsupervised setting of the VAE and MMD-VAE to learn the LFs of the DNA methylation data of 886 samples (GDC cohort). We have selected the first 2 LFs of the 128 LFs to cluster the samples into two groups (cancer and normal). The two-dimensional embedding of the DNA methylation dataset’s input features was plotted on scatter plots for PCA, t-SNE, VAE and MMD-VAE. As illustrated in Fig. 2, even with the unsupervised setting, all the dimensionality reduction methods demonstrate clustering accuracy over 95%, thanks to the discriminative nature of the input features. MMD-VAE outperforms others by correctly clustering 883 samples out of 886. However, the distance between the clusters is an issue, especially in MMD-VAE, which was improved (shown in Fig. 2e,f) for VAE and MMD-VAE by combining t-SNE with them. The cancer samples are compact within the cluster (orange dots) compared to the normal samples. The sub-clusters within the normal samples could be due to the variances within the samples.
- Molecular subtypes clustering: We clustered the transcriptional subtypes using the LFs learned through unsupervised and supervised VAE and MMD-VAE models. For the LFs learned via unsupervised model, we have selected the first 2 LFs of the learned 128 LFs to cluster the molecular subtypes. The two-dimensional embedding of the mono omic, di-omics and tri-omics datasets’ input features were plotted on scatter plots for PCA, t-SNE, VAE and MMD-VAE. Figure 9 in Supplementary documents presents the results of 2 LFs-based molecular subtypes clustering. As seen in Fig. 9b–i in Supplementary documents, all the dimensionality reduction methods poorly clustered the samples into four subtypes using the mono- and tri-omics datasets. This result is expected as the original omics datasets are not discriminative or well representative of the transcriptional subtypes. As Fig. 9a in Supplementary documents illustrates, even the most relevant transcriptional dataset (mRNA) do not represent the transcriptional subtypes. Hence, the unsupervised PCA, t-SNE, VAE and MMD-VAE models struggle to cluster the transcriptional subtypes. In this context, we can use the supervised versions of these models, especially VAE and MMD-VAE. We used the supervised VAE and MMD-VAE models to learn the task-oriented (i.e., the transcriptional subtypes) or guided LFs from the mono-, di- and tri-omics datasets. We have selected the first 2 LFs of the learned 128 LFs to cluster the molecular subtypes. Figure 3 presents a part of the clustering results for the supervised VAE and MMD-VAE. As Fig. 3a–j illustrates, the supervised VAE and MMD-VAE have significantly improved their clustering performance compared to their unsupervised counterparts (Fig. 9 in Supplementary documents) in all omics datasets. As illustrated in the Figure, the transcriptional (mRNA -mono-omics) dataset is outperforming other datasets, mainly other mono- omics (i.e., methylation and CNV) datasets, and MMD-VAE outperforms VAE in most datasets. Also, we have combined the t-SNE with VAE and MMD-VAE, which improve the performance (shown in Fig. 3k,l compared to their implementations without t-SNE.

**Table 2:** Molecular subtypes classification performances using LFs learned via supervised VAE/MMD-VAE.

Method	Omics_data	Accuracy	Precision	Recall	f1 score
V-VAE	CNV	58.3 ± 0.3	0.63 ± 0.01	0.58 ± 0.03	0.579 ± 0.03
MMD-VAE	CNV	54.3 ± 0.31	0.58 ± 0.02	0.54 ± 0.02	0.53 ± 0.03
V-VAE	mRNA	95.7 ± .5	0.95 ± 0.008	0.95 ± 0.05	0.95 ± 0.006
MMD-VAE	mRNA	93.8 ± .97	0.93 ± 0.006	0.93 ± 0.005	0.93 ± 0.006
V-VAE	Methylation	72.3 ± .8	0.73 ± 0.02	0.72 ± 0.009	0.71 ± 0.006
MMD-VAE	Methylation	75.2 ± .9	0.75 ± 0.019	0.75 ± 0.018	0.75 ± 0.015
V-VAE	CNV_mRNA	93.7 ± .27	0.93 ± 0.01	0.93 ± 0.008	0.93 ± 0.007
MMD-VAE	CNV_mRNA	93.7 ± .37	0.94 ± 0.006	0.93 ± 0.007	0.93 ± 0.007
V-VAE	mRNA_methylation	87.1 ± 1.1	0.87 ± 0.009	0.87 ± 0.008	0.87 ± 0.005
MMD-VAE	mRNA_methylation	93.2 ± .97	0.93 ± 0.02	0.93 ± 0.008	0.93 ± 0.005
V-VAE	CNV_mRNA_methylation	89.4 ± .6	0.89 ± 0.02	0.89 ± 0.006	0.89 ± 0.004
MMD-VAE	CNV_mRNA_methylation	95.5 ± .37	0.95 ± 0.02	0.95 ± 0.008	0.95 ± 0.009

#### Classification

- Cancer samples identification: We used an SVM-based classifier to identify the cancer samples from the normal samples using the LFs learned through the unsupervised PCA, t-SNE, VAE and MMD-VAE. Table 3 in Supplementary documents presents the classification performances for the DNA methylation dataset of 886 samples (GDC cohort). All the models except t-SNE have more than 99% classification accuracy with very high precision (0.99), recall (0.99) and f1 score (0.99). The discriminative features (cancer vs normal) of the DNA methylation data is the main reason for this classification performance. Molecular subtypes classification: Like the transcriptional subtypes clustering, we used the LFs learned through the unsupervised and supervised VAE and MMD-VAE models in molecular subtypes classification. For the unsupervised setting, we also compared the classification performance of the LFs learned through VAE and MMD-VAE with of the LFs learned through PCA and t-SNE. Table 4 in Supplementary documents presents the classification performance of an ANN-based classifier utilising the LFs learned via these unsupervised models from mono-, di- and tri-omics datasets. As we can see from the table, the classifier using the PCA and t-SNE generated LFs poorly classify the existing transcriptional subtypes in all omics datasets. On the other hand, the classifier using the VAE and MMD-VAE generated LFs can classify the transcriptional subtypes for mono-omics (mainly mRNA), di- and tri-omics data with higher accuracies in the range of 73.2–81.44%. However, the performances may not be acceptable in many real-life applications. Lack of discriminative features within the omics datasets for the transcriptional subtypes is the main reason for the low accuracies. Supervised learning of the LFs can improve the classification performance. In the supervised setting, VAE or MMD-VAE and the classifier jointly learn the LFs using the transcriptional subtypes as the supervisory guidance. We trained the joint models on the mono-, di- and tri-omics datasets and tested the models separately. Table 2 presents the performances of the molecular subtypes classification in terms of accuracy, precision, recall and f1 score. Figure 10 in Supplementary documents presents the confusion matrices for few of these classification tasks. As presented in the table, the molecular subtypes classification performances have significantly improved in all matrices (i.e., accuracy, precision, recall and f1 score) compared to the unsupervised VAE/MMD-VAE (Table 4 in Supplementary documents). For example, except the CNV and methylation datasets, MMD-VAE and VAE respectively show accuracies in the range of 93.2-95.5%, and 87.1-95.7% with high precision, recall and f1 scores. The performances of the CNV and DNA methylation are not satisfactory as they are not transcriptional omics data. Even these non-transcriptional datasets, especially the DNA methylation dataset, show a good classification performance with an accuracy range 72.3–75.2%. This performance could be due to the association or correlation between the omics datasets^55–58^. For the same reason, the use of integrated non-transcriptional and transcriptional data helps to maintain a similar performance or improve the performances of the transcriptional subtypes clustering and classification. For example, the accuracy of MMD-VAE using mRNA is 93.8%, which has been maintained (93.7%) in case of the integrated CNV-mRNA, and improved to 95.5% in case of the integrated CNV-mRNA-methylation datasets. The confusion matrices in Fig. 10b,d,f in Supplementary documents for MMD-VAE illustrate similar results. As a dimensionality reduction algorithm, in majority datasets, MMD-VAE shows better performance compared to VAE. This performance difference could be due to the MMD-based loss function. We tested the supervised MMD-VAE and VAE on omics datasets with three different sample sizes (i.e., 292, 459, and 481) to demonstrate our findings’ robustness. We used the learned LFs to classify the transcriptional subtypes and presented the classification accuracies in Table 5 in Supplementary documents. As seen in the Table, both VAE and MMD-VAE show robust classification accuracy for the same omics data with different sample sizes.

The dimensionality reduction performance results of VAE and MMD-VAE in clustering and classification demonstrate the followings:

- in any downstream analysis (e.g., classification) unsupervised dimensionality reduction is useful if the input dataset is discriminative (e.g., cancer vs normal samples), otherwise supervised dimensionality reduction is necessary, and
- integrated dimensionality reduction and multi-omics analysis of data may improve or maintain the similar performance of their mono-omics counterparts exploiting their association, without confounding each other.

### Survival analysis

**Figure 4:**
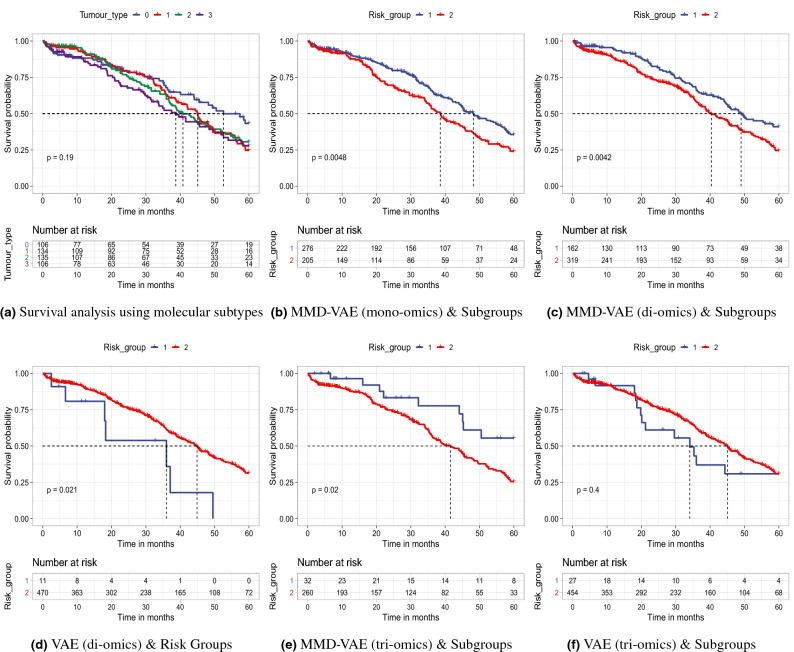
Survival analysis using existing using molecular subtypes and CRLFs-based survival subgroups: (**a**) survival analysis using the existing transcriptional subtypes show that they are not linked to the survival ($p =0.19<0.05$), (**b**–**f**) survival analysis using the two subgroups show significant survival differences ($p <0.05$) between the groups. The results in (**e**) for 292 samples, and the rest are for 481 samples.

We did a comprehensive survival analysis using eleven datasets, including mono-omics and multi-omics data, particularly for the samples with existing transcriptional subtypes and inferred survival/risk groups. Considering the space limitation, we presented a subset but enough of the results (Fig. 4 and Fig. 12, and Table 7 in Supplementary documents) that significantly represent the performance of VAE and MMD-VAE in survival analysis. Figure 4a (for 481 samples) and Fig. 11 in Supplementary documents (for 292 samples) present the Kaplan-Meier survival curves for existing transcriptional subtypes. The subtypes are not clinically significant or associate with survival of patients/samples (log-rank $p >0.05$) (Fig. 4a and Fig. 11 in Supplementary documents).

For LFs-based survival analysis, we conducted a univariate Cox-PH regression on each of the 128 LFs from each dataset. We identified 5-22 CRLFs associated with survival. The number of CRLFs is different for each omics dataset. For example, we found 22 LFs for CNV dataset and only 5 LFs for the integrated CNV, DNA methylation and mRNA dataset). We did a two-stage survival analysis of the samples (481 and 292) using the two inferred subgroups. In the first stage, we plotted Kaplan-Meier survival curves for all the samples. As seen in the Kaplan-Meier survival curves (Fig. 4b–f) of the inferred groups by VAE and MMD-VAE, there is a significant survival differences (log-rank $p>0.05$) for all the omic(s) data accept the tri-omics (log-rank $p = 0.4$ is higher than threshold $\alpha = 0.05$), especially for the VAE. This results could be due to the uninformative LFs learned by the VAE.

In the second stage, we predicted survival subgroup labels using an SVM-based classifier splitting the samples into training and test data using a 60/40 split ratio. After predicting survival groups for the test datasets, we ran two multivariate Cox-PH regressions (one for clinical and one for combined = subgroup + clinical co-variates) on the training samples, then predicted survival using the labels of the test datasets. For the clinical co-variates, we considered three clinicopathological characteristics of the considered patients: (i) age at diagnosis, (ii) clinical or FIGO stage, and (iii) grade. We calculated C-indexes, Brier scores and models’ p-values for the training and held-out test samples for the multivariate Cox-PH regressions. As seen in Table 7 in Supplementary documents, the training samples generated moderately high C-indexes in between $0.62 -0.68$, low Brier scores in between $0.17-0.19$ with significant log-rank p-values $<0.05$ of the Cox-PH model. A similar trend is observed for the held-out datasets with little lower C-indexes ($0.60-0.66$) and little higher Brier scores ($0.19-0.23$) with significant log-rank p-values $<0.05$ of the Cox-PH model. Importantly, as seen from the Table 7 in Supplementary documents the performances of VAE and MMD-VAE have been improved in case of combined survival analysis compared to only clinical variables. This confirm that identified survival subgroup does not confound with clinicopathological variables, rather it improves the prognosis. Finally, we have plotted Kaplan-Meier survival curves for the predicted survival group labels. As shown in Fig. 12a–f in Supplementary documents, similar to Fig. 4b–f, there is a significant survival differences (log-rank $p>0.05$) for all the omic(s) between predicted survival groups for all (presented) omics data. However, few datasets’ *p* values are higher than the threshold (0.05). Potential reasons for the higher *p* values or insignificant differences between the predicted survival groups for the datasets could be (i) the smaller sample size with few events to identify the differences and (ii) too much compression may have obscured the clinically relevant features.

From Fig. 4b–f and Fig. 12 and Table 7 in Supplementary documents we have the following two key observations:

- Impact of inferred subgroups on survival: In case of mono-omics and multi-omics data, inferred subgroups combined with the clinical covariates (i.e., stage, grade and age) improve the survival prediction. For example, subgroups learned using the MMD-VAE on CNV mono-omics data combined with clinical features shows higher C-index value (0.63) than the C-index value (0.62) for the clinical features. However, the improvement is not that significant. In summary, inferred subgroups, combined with the clinical features, demonstrate survival prediction performance higher than or similar to the clinical features.
- mono-omics vs multi-omics based LFs in survival: Similar to molecular subtypes classification, multi-omics based LFs may incrementally (e.g., di- and tri-omics) improve the survival subgroups classification accuracy compared to mono-omics based LFs (shown in Table 6 in Supplementary documents). However, multi-omics (i.e., CNV_mRNA, CNV_mRNA_methylation) based LFs and subgroups predicted based on them do not improve the survival prediction performances than their mono-omics counterparts. Potential reasons for the lower performance using multi-omics based LFs could be due to (i) smaller sample size with few events (e.g., 143 out of 292 samples) to identify the differences, (ii) too much compression (e.g., for CNV + DNA methylation + mRNA data 64,398 input features to 128 LFs) may have obscured the clinically relevant features.

**Figure 5:**
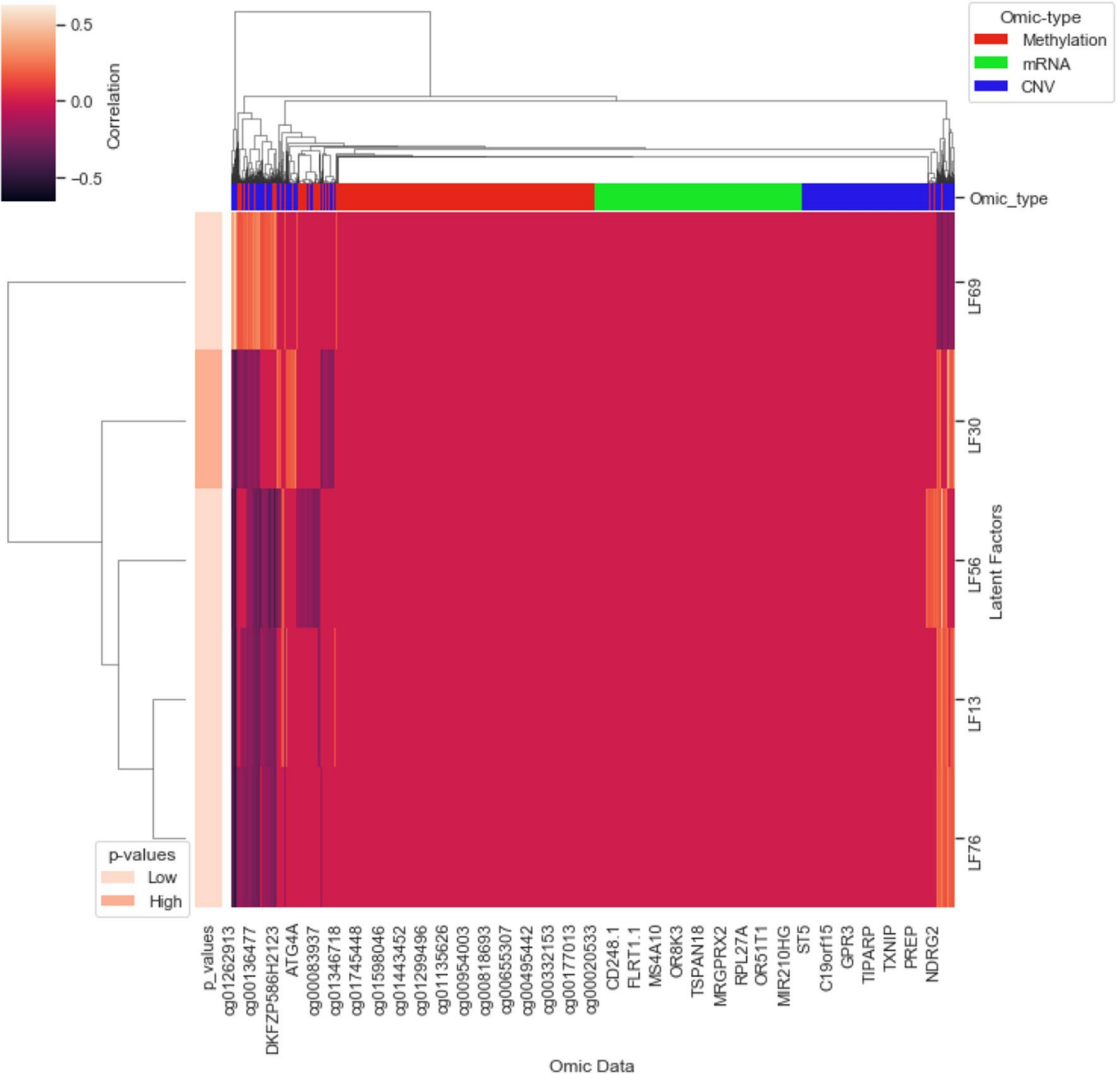
Association between CRLFs and input features: Input features of CNV_mRNA_methylation omics data are clustered based on the correlation data with the identified CRLFs. For example, the NDRG2 gene has strong correlation with LF30 and LF69.

Finally, we presented a simple method to identify potential prognostic biomarkers from the clinically relevant LFs using a linear model. Figure 5 presents the association between the CRLFs and the input features for tri-omics (CNV_mRNA_methylation) data. For example, our mapping has identified the NDRG2 gene as a potential biomarker for ovarian cancer^69^. Thus, we can identify the other responsible gene or cgp island that could jointly work as a multi-omics prognostic biomarker. However, the linear mapping is not the best solution to identify a set of biomarkers from the learned CRLFs as it is only identifying correlations values within $\pm\,0.5$. We need further research in this direction.

## Conclusion

Integrated multi-omics data analysis of cancer is vital for a comprehensive understanding of cellular functions, responsible for cancer and its development. DL algorithms have become a useful and popular tool for integrated multi-omics analysis of cancer data in recent years. However, imbalanced high dimensional multi-omics data makes a DL based integrated multi-omics analysis difficult, especially in individual cancer. DL based dimensionality reduction techniques, such as variational autoencoder is a potential solution to this dimensionality issue. In this work, we have designed and developed VAE and MMD-VAE based dimensionality reduction techniques for mono-omics and multi-omics data analysis of cancer. These techniques can learn latent features from any omics data using unsupervised and supervised training process. Then, we used the developed techniques to learn latent features from the mono-omics, di-omics and tri-omics datasets of ovarian cancer. Finally, we used the learned latent features to analyse ovarian cancer through cancer samples identification, molecular subtypes clustering and classification and survival analysis. The results, especially subtype clustering and classification and survival subgroups prediction demonstrate that integrated di- and tri-omics based LFs can perform better or similar to their mono-omics counterparts based LFs. Also, molecular subtypes clustering and classification results show that MMD-VAE is outperforming VAE in most datasets. Notably, the results demonstrate that unsupervised dimensionality reduction is useful in the downstream analysis (e.g., classification) when the input dataset is discriminatory, otherwise supervised dimensionality reduction is needed. Finally, inferred and predicted survival subgroups results show a significant survival difference between the two subgroups. However, multi-omics based LFs and subgroups predicted based on them do not improve the survival prediction performances than their mono-omics counterparts. One of the potential reasons for this could be a sub-optimal number of LFs or dimension of the selected dimension space. Moreover, the straightforward integration (concatenation based) strategy, treating omics measurements from different platforms equally and performing integration in a parallel fashion may not always be useful. The linear model used for mapping between CRLFs and input features may not be the best solution for biomarkers identification. There is significant scope for future work in these areas.

The residual disease is one of the strongest predictors for outcome estimation after any cancer therapy or treatment. Hence, VAE or MMD-VAE based integrated multi-omics analysis on predicting residual disease for ovarian cancer will be one of our future works.

## Supplementary Information

Supplementary Information.
