# Author Correction: Integrated multi‑omics analysis of ovarian cancer using variational autoencoders

**DOI:** 10.1038/s41598-021-95882-y

**Published:** 2021-08-11

**Authors:** Muta Tah Hira, M. A. Razzaque, Claudio Angione, James Scrivens, Saladin Sawan, Mosharraf Sarker

**Affiliations:** 1 School of Health and Life Sciences, Teesside University, Middlesbrough, TS4 3BX UK; 2 School of Computing, Eng. & Digital Tech., Teesside University, Middlesbrough, TS4 3BX UK; 3 The James Cook University Hospital, Middlesbrough, TS4 3BW UK

Correction to: *Scientific Reports* https://doi.org/10.1038/s41598-021-85285-4, published online 18 March 2021

The original version of this Article contained an error in the spelling of the author Mosharraf Sarker which was incorrectly given as Mosharraf Sarkar.

The original Article has been corrected.

