# Supplementary material for: Integrated multi-omics analysis of ovarian cancer using variational autoencoders
Source: Sci Rep. 2021 Mar 18;11:6265. doi: 10.1038/s41598-021-85285-4 (PMC7973750; doi:10.1038/s41598-021-85285-4)
Supplement: Supplementary file 1 — Supplementary Information. [file 41598_2021_85285_MOESM1_ESM.pdf]

Supplementary Documents

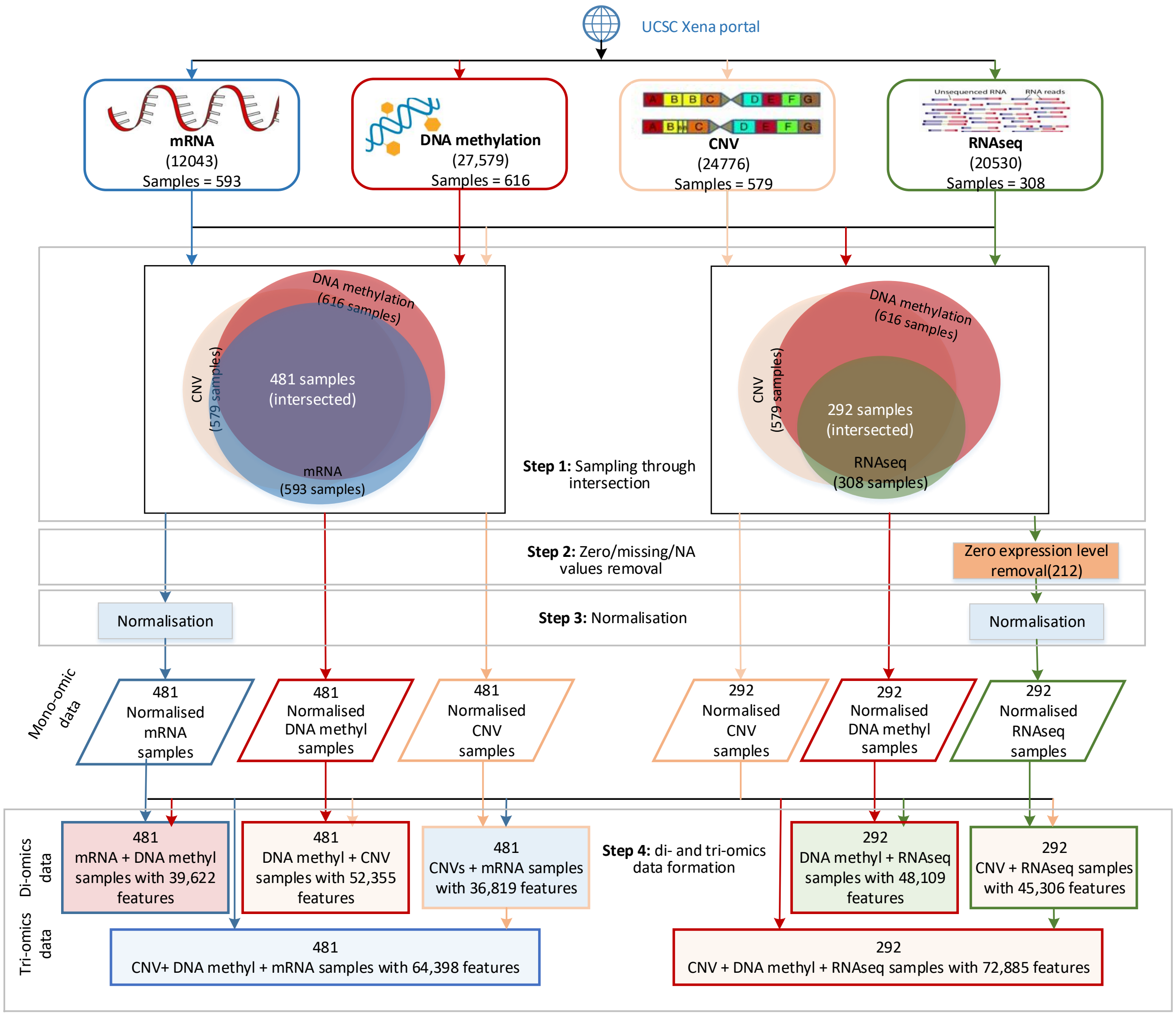

Figure 6. Data prepossessing with di- and tri-omics data generation. Uses a 4-steps process: first 3 steps (intersection, zero/missing/NA removal, and normalisation) for preprocessing, and step 4 is to generate di- and tri-omics data concatenating the corresponding mono omic datasets.

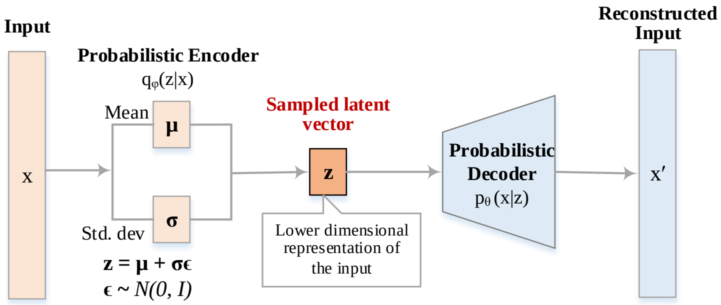

Figure 7. A basic architecture of a VAE that includes a probabilistic encoder that encodes the input x and a probabilistic decoder that decodes x' from latent vector z.

Table 3. Cancer identification performance of the LFs learned using the unsupervised methods

Method	Omics_data	Accuracy	Precision	Recall	f1 score
PCA + SVM	methylation	99.10±1.22%	0.992±0.011	0.991±0.012	0.991±0.012
t-SNE + SVM	methylation	93.91±2.20%	0.940±0.023	0.939±0.022	0.938±0.022
VAE + SVM	methylation	99.10±0.98%	0.992±0.009	0.991±0.010	0.991±0.010
MMD-VAE + SVM	methylation	99.44±0.76%	0.995±0.007	0.994±0.008	0.994±0.008

Table 4. Ovarian cancer molecular subtypes classification performances (unsupervised LFs)

Method	Omics_data	Accuracy	Precision	Recall	f1 score
PCA + ANN	CNV	42.27±0.1%	0.393±0.01	0.423±0.01	0.358±0.01
t-SNE + ANN	CNV	36.08±0.17%	0.426±0.016	0.361±0.01	0.286±0.01
V-VAE + ANN	CNV	49.90±0.51%	0.493±0.010	0.499±0.05	0.481±0.01
MMD-VAE + ANN	CNV	52.58±0.40%	0.523±0.01	0.526±0.04	0.489±0.01
PCA + ANN	mRNA	57.73±0.20%	0.451±0.01	0.577±0.01	0.505±0.01
t-SNE + ANN	mRNA	64.54±0.82%	0.698±0.01	0.645±0.008	0.608±0.008
V-VAE + ANN	mRNA	81.44±0.40%	0.822±0.03	0.814±0.029	0.814±0.028
MMD-VAE + ANN	mRNA	78.35±0.390%	0.785±0.03	0.784±0.026	0.781±0.027
PCA + ANN	CNV_mRNA	32.99±0.31%	0.271±0.02	0.330±0.020	0.266±0.019
t-SNE + ANN	CNV_mRNA	38.14±0.29%	0.299±0.03	0.381±0.010	0.325±0.02
V-VAE + ANN	CNV_mRNA	73.20±0.22%	0.727±0.02	0.732±0.000	0.726±0.000
MMD-VAE + ANN	CNV_mRNA	81.44±0.20%	0.817±0.01	0.814±0.000	0.813±0.000
PCA + ANN	CNV_mRNA_methylation	37.11±0.45%	0.413±0.02	0.371±0.000	0.342±0.000
t-SNE + ANN	CNV_mRNA_methylation	38.14±0.39%	0.561±0.03	0.381±0.000	0.344±0.000
V-VAE + ANN	CNV_mRNA_methylation	78.35±0.2%	0.784±0.01	0.784±0.001	0.784±0.01
MMD-VAE + ANN	CNV_mRNA_methylation	80.21±0.19%	0.808±0.03	0.802±0.02	0.802±0.02

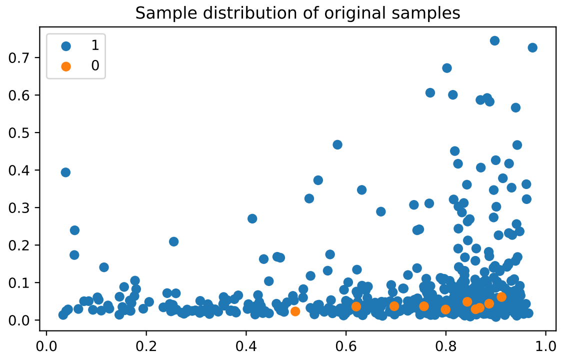

(a) Original DNA methylation dataset

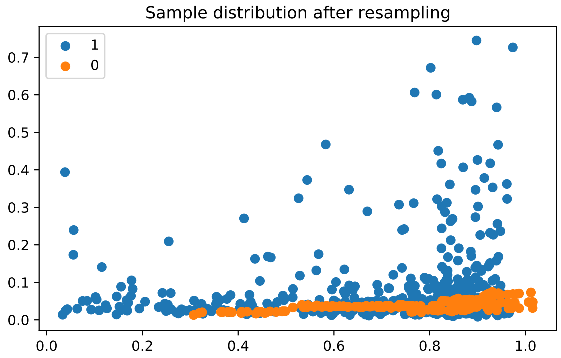

(b) Re-sampled DNA methylation dataset

Figure 8. Distribution of normal and cancer samples in DNA methylation data from the GDC TCGA Ovarian Cancer (OV) (44):(a) the original dataset with 613 samples is highly imbalanced with 10 normal samples compared to the 603 cancer samples (class ratio: 1.36: 98:64), (b) Re-sampled datasets with 886 samples and with class ratio 31.94:68.06 between the normal and cancer samples. Legends: 0- Normal, 1- Cancer.

Table 5. Molecular subtypes classification accuracies for different sample sizes

Sample size	Method	Omics type	Accuracy
292	VAE	mono	93.1
292	MMD-VAE	mono	94.3
292	VAE	tri	88.5
292	MMD-VAE	tri	94.5
459	VAE	mono	93.7
459	MMD-VAE	mono	92.3
459	VAE	tri	89.3
459	MMD-VAE	tri	95.3
481	VAE	mono	95.7
481	MMD-VAE	mono	93.8
481	VAE	tri	89.4
481	MMD-VAE	tri	95.5

Table 6. Classification accuracy of survival subgroups

Omic (s) data	Algorithm	Dataset	Classification Accuracy
mRNA (mono-omics)	MMD	Training	1
mRNA (mono-omics)	MMD	Test	0.84
mRNA_methylation (di-omics)	MMD	Training	0.99
mRNA_methylation (di-omics)	MMD	Test	0.99
CNV_mRNA_methylation (tri-omics)	MMD	Training	1
CNV_mRNA_methylation (tri-omics)	MMD	Test	1

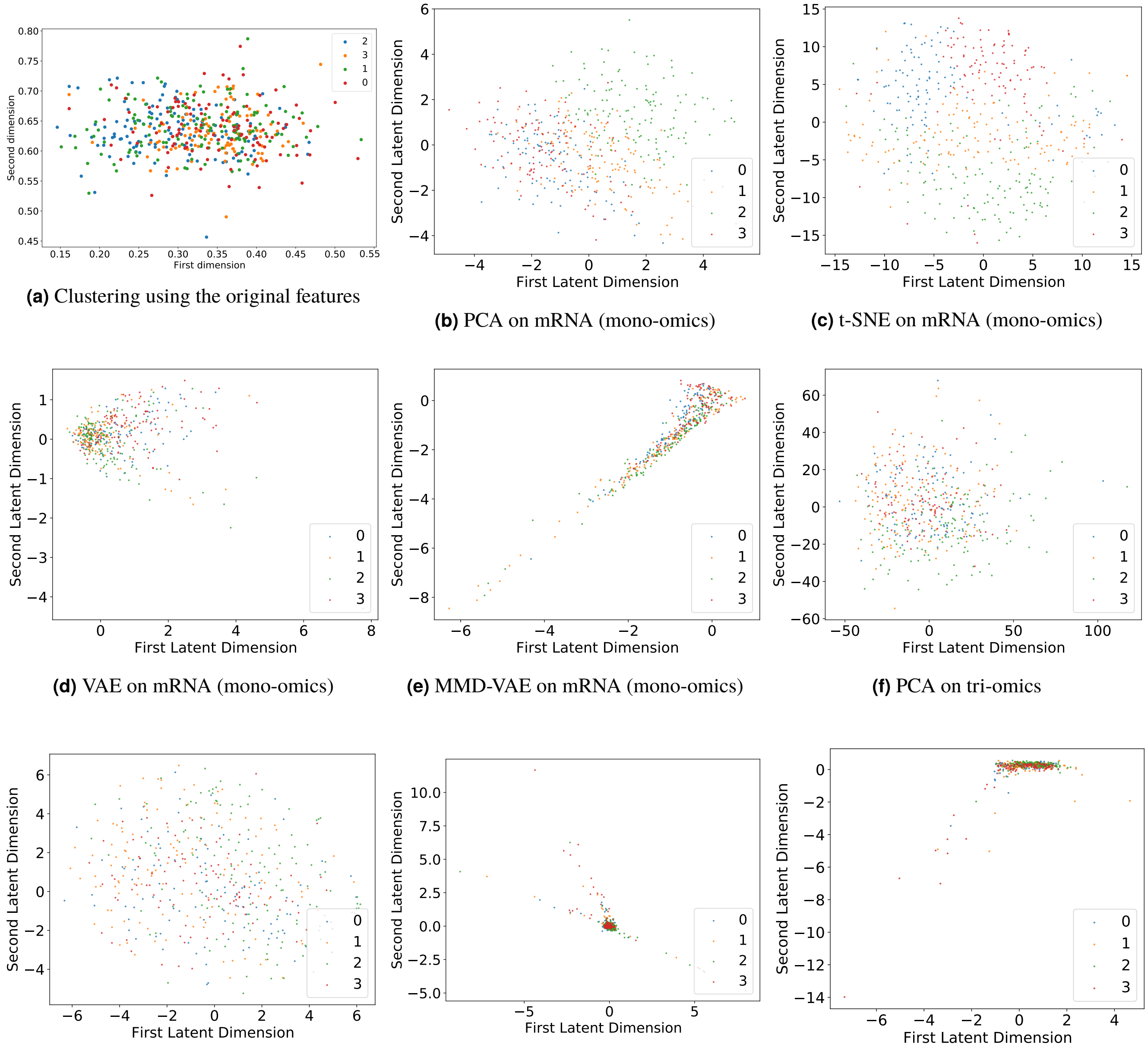

(a) Clustering using the original features

(b) PCA on mRNA (mono-omics)

(c) t-SNE on mRNA (mono-omics)

(d) VAE on mRNA (mono-omics)

(e) MMD-VAE on mRNA (mono-omics)

(f) PCA on tri-omics

(g) t-SNE on tri-omics

(h) VAE on tri-omics

(i) MMD-VAE on tri-omics

Figure 9. Molecular subtypes clustering using PCA, t-SNE, VAE, and MMD-VAE (2D or 2 LFs): Figure (a) represents the clustering of subtypes using the original mRNA. Figures (b)-(i) respectively present the subtypes clustering using the LFs learned from the mono-omics and tri-omics data throuhg PCA, t-SNE, VAE, and MMD-VAE. Legends: 0- Immunoreactive, 1- Differentiated, 2- Proliferative and 3- Mesenchymal.

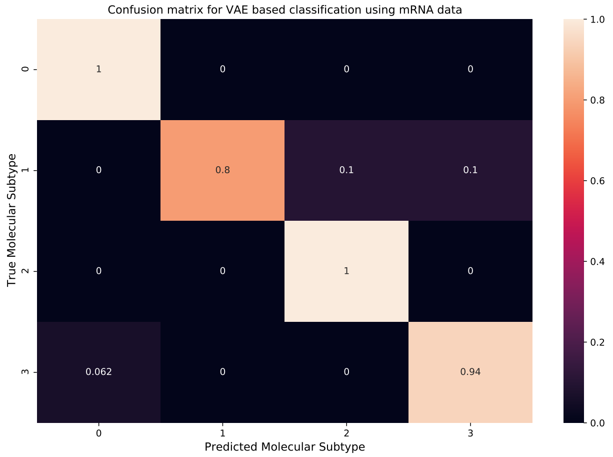

(a) CM for classification using VAE (mono-omics)

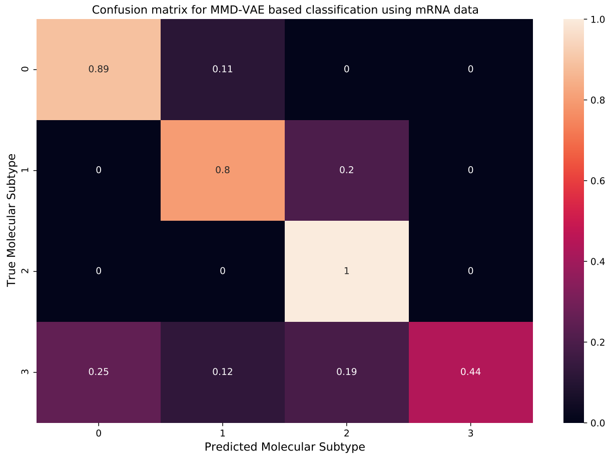

(b) CM for classification using MMD-VAE (mono-omics)

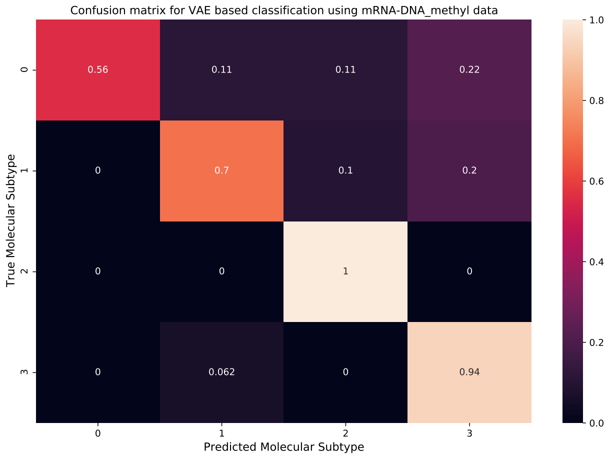

(c) CM for classification using VAE (di-omics)

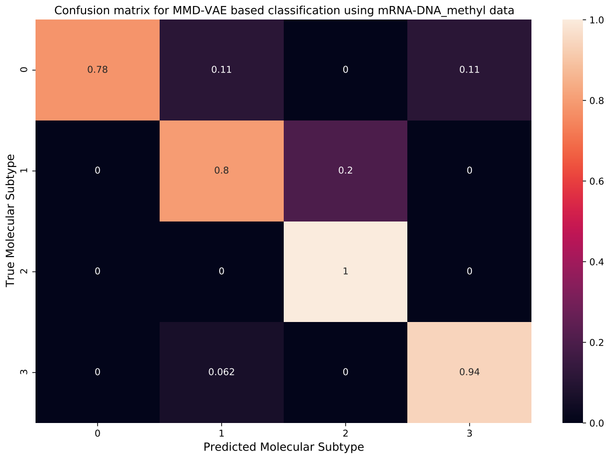

(d) CM for classification using MMD-VAE (di-omics)

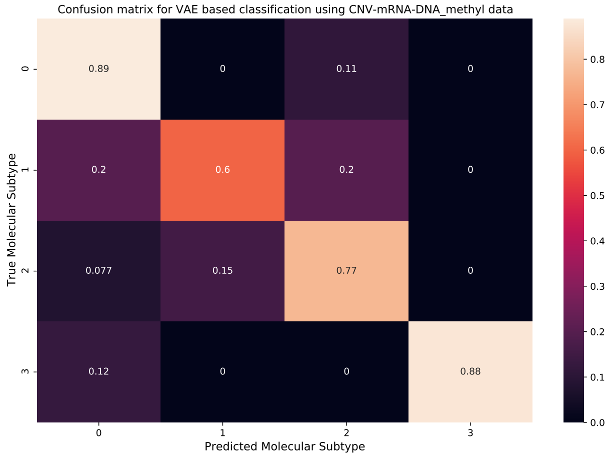

(e) CM for classification using VAE (tri-omics)

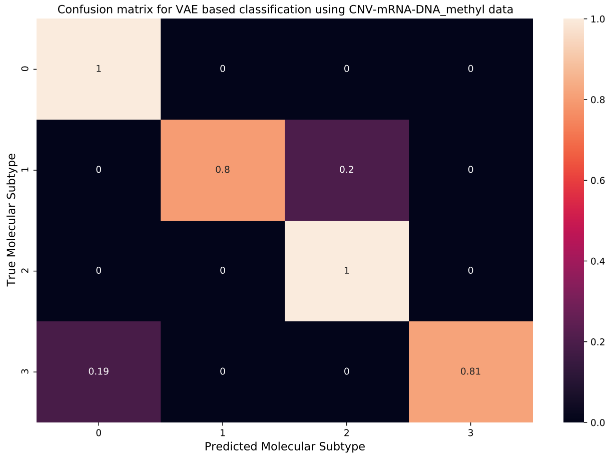

(f) CM for classification using MMD-VAE (tri-omics)

Figure 10. Confusion matrices of molecular subtypes using supervised VAE & MMD-VAE: (a)-(d) for mono-omics, (e) - (f) for di-omics and (g-h) tri-omics data. Legends: 0- Immunoreactive, 1- Differentiated, 2- Proliferative and 3- Mesenchymal, and CM- confusion matrix.

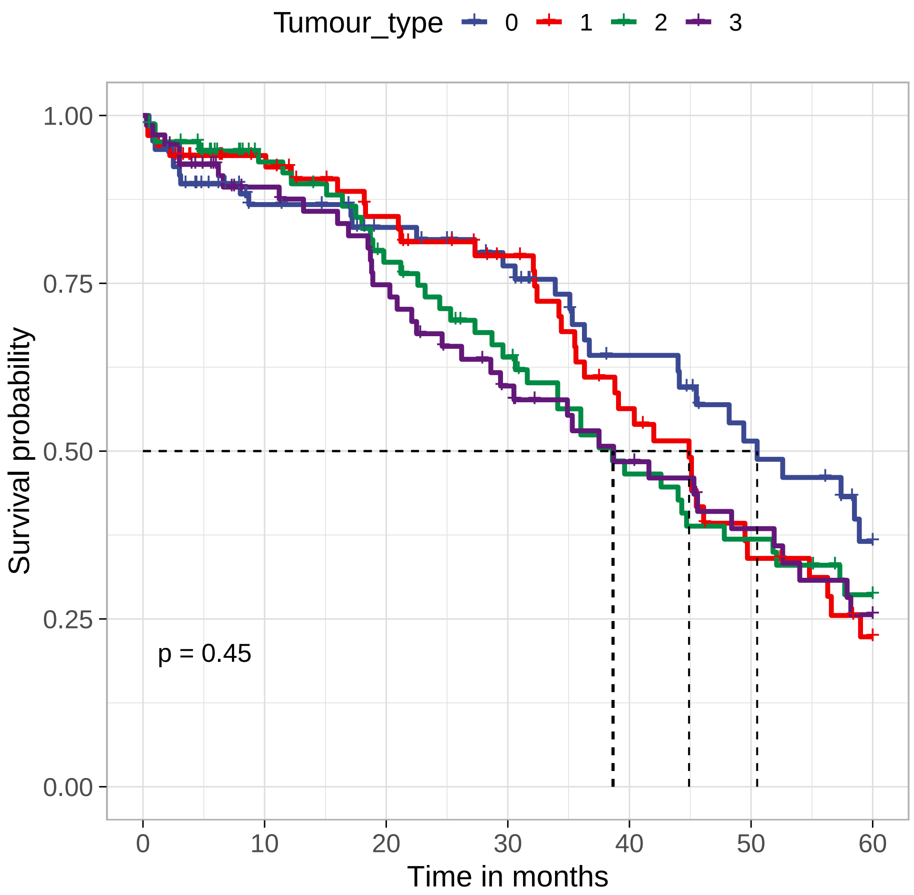

Figure 11. Survival analysis of existing molecular subtypes (292 samples)

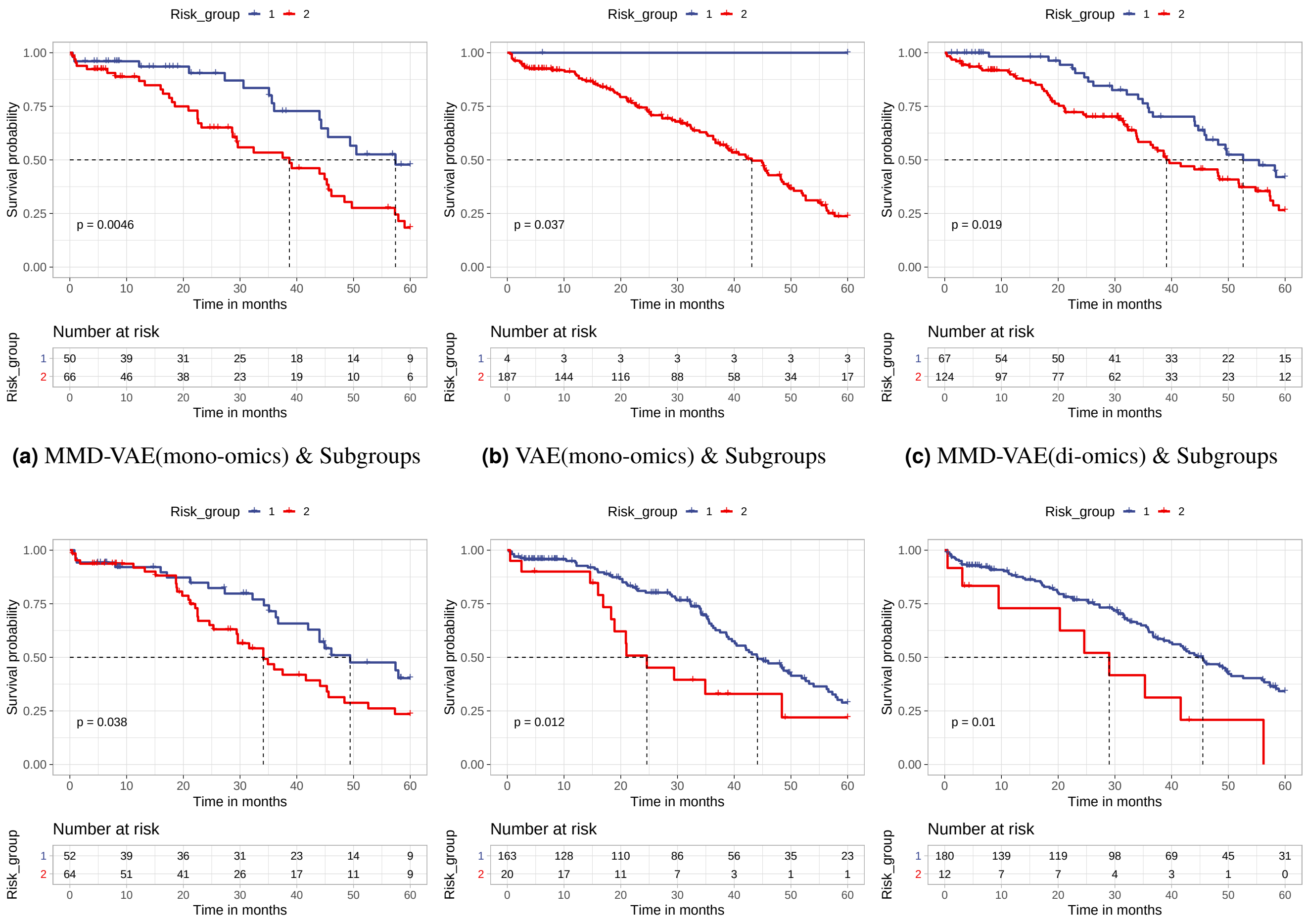

(a) MMD-VAE(mono-omics) & Subgroups **(b)** VAE(mono-omics) & Subgroups **(c)** MMD-VAE(di-omics) & Subgroups

(d) MMD-VAE(di-omics) & Subgroups **(e)** VAE (di-omics) & Subgroups **(f)** MMD-VAE (tri-omics) & Subgroups

Figure 12. Predicted survival analysis using CRLFs-based subgroups: (a-f) survival analysis using the predicted subgroups show significant survival differences ($p < .05$) between the groups. The results in Figure (a) and (d) for 292 samples, and the rest are for 481 samples.

Table 7. Performance of the DL architecture for VAE and MMD-VAE in survival prediction

Omic-type	Algorithm	Co-variates	Dataset	C-index	Brier score	p-value
CNV	MMD	Clinical	Training	0.64	0.194	0.003
CNV	MMD	Clinical	Test	0.62	0.19	0.003
CNV	MMD	Clinical + subgroup	Training	0.65	0.19	0.0006
CNV	MMD	Clinical + subgroup	Test	0.63	0.192	0.0006
CNV	VAE	Clinical	Training	0.65	0.186	0.0015
CNV	VAE	Clinical	Test	0.64	0.23	0.0015
CNV	VAE	Clinical + subgroup	Training	0.68	0.18	0
CNV	VAE	Clinical + subgroup	Test	0.66	0.24	0
mRNA	MMD	Clinical	Training	0.65	0.18	0.006
mRNA	MMD	Clinical	Test	0.62	0.23	0.006
mRNA	MMD	Clinical + subgroup	Training	0.67	0.18	0.006
mRNA	MMD	Clinical + subgroup	Test	0.60	0.23	0.006
mRNA	VAE	Clinical	Training	0.64	0.19	0.015
mRNA	VAE	Clinical	Test	0.63	0.18	0.015
mRNA	VAE	Clinical + subgroup	Training	0.63	0.19	0.017
mRNA	VAE	Clinical + subgroup	Test	0.62	0.18	0.017
CNV_mRNA	MMD	Clinical	Training	0.67	0.177	0.00
CNV_mRNA	MMD	Clinical	Test	0.60	0.20	0.24
CNV_mRNA	MMD	Clinical + subgroup	Training	0.68	0.19	0.00003
CNV_mRNA	MMD	Clinical + subgroup	Test	0.64	0.20	0.09
CNV_mRNA	VAE	Clinical	Training	0.62	0.184	0.005
CNV_mRNA	VAE	Clinical	Test	0.65	0.193	0.045
CNV_mRNA	VAE	Clinical + subgroup	Training	0.63	0.183	0.0085
CNV_mRNA	VAE	Clinical + subgroup	Test	0.65	0.193	0.09
CNV_mRNA_methylation	MMD	Clinical	Training	0.61	0.20	0.038
CNV_mRNA_methylation	MMD	Clinical	Test	0.65	0.22	0.042
CNV_mRNA_methylation	MMD	Clinical + subgroup	Training	0.64	0.19	0.01
CNV_mRNA_methylation	MMD	Clinical + subgroup	Test	0.66	0.23	0.08
CNV_mRNA_methylation	VAE	Clinical	Training	0.62	0.19	0.026
CNV_mRNA_methylation	VAE	Clinical	Test	0.64	0.197	0.099
CNV_mRNA_methylation	VAE	Clinical + subgroup	Training	0.62	0.188	0.083
CNV_mRNA_methylation	VAE	Clinical + subgroup	Test	0.63	0.195	0.10
